# Sustainment of the TeleSleep program for rural veterans

**DOI:** 10.3389/frhs.2023.1214071

**Published:** 2023-11-10

**Authors:** Jeffrey K. Belkora, Linda Ortiz DeBoque, Robert L. Folmer, Annette M. Totten, Katherine Williams, Mary A. Whooley, Eilis Boudreau, Charles W. Atwood, Michelle Zeidler, Talayeh Rezayat, Priyanka Chilakamarri, Kathleen F. Sarmiento

**Affiliations:** ^1^ San Francisco VA Health Care System, San Francisco, CA, United States; ^2^ Institute for Health Policy Studies and Department of Surgery, University of California, San Francisco, San Francisco, CA, United States; ^3^ VA Portland Health Care System, Portland, OR, United States; ^4^ Department of Otolaryngology, Oregon Health & Science University, Portland, OR, United States; ^5^ Department of Medical Informatics and Clinical Epidemiology, Oregon Health & Science University, Portland, OR, United States; ^6^ Measurement Science Quality Enhancement Research Initiative, San Francisco VA Health Care System, San Francisco, CA, United States; ^7^ Department of Medicine, University of California, San Francisco, San Francisco, CA, United States; ^8^ Department of Medical Informatics and Clinical Epidemiology, Oregon Health & Science University, Portland, OR, United States; ^9^ Department of Neurology, Oregon Health & Science University, Portland, OR, United States; ^10^ Pulmonary Section and Sleep Medicine, VA Pittsburgh Healthcare System, Pittsburgh, PA, United States; ^11^ Division of Pulmonary, Allergy, Critical Care Medicine, UPMC and University of Pittsburgh, Pittsburgh, PA, United States; ^12^ Pulmonary, Critical Care, and Sleep Medicine, VA Greater Los Angeles Health Care System, Los Angeles, CA, United States; ^13^ Department of Medicine, University of California, Los Angeles, Los Angeles, CA, United States; ^14^ Department of Medicine, University of Nevada, Reno, NV, United States; ^15^ Department of Neurology, University of California, San Francisco, San Francisco, CA, United States

**Keywords:** implementation science, maintenance, sustainment, telemedicine, sleep medicine

## Abstract

**Background:**

In fiscal year 2021, the Veterans Health Administration (VHA) provided care for sleep disorders to 599,966 Veterans, including 189,932 rural Veterans. To further improve rural access, the VA Office of Rural Health developed the TeleSleep Enterprise-Wide Initiative (EWI). TeleSleep's telemedicine strategies include tests for sleep apnea at the Veteran's home rather than in a sleep lab; Clinical Video Telehealth applications; and other forms of virtual care. In 2017 and 2020, VHA provided 3-year start-up funding to launch new TeleSleep programs at rural-serving VA medical facilities.

**Methods:**

In early 2022, we surveyed leaders of 24 sites that received TeleSleep funding to identify successes, failures, facilitators, and barriers relevant to sustaining TeleSleep implementations upon expiration of startup funding. We tabulated frequencies on the multiple choice questions in the survey, and, using the survey's critical incident framework, summarized the responses to open-ended questions. TeleSleep program leaders discussed the responses and synthesized recommendations for improvement.

**Results:**

18 sites reported sustainment, while six were “on track.” Sustainment involved medical centers or regional entities incorporating TeleSleep into their budgets. Facilitators included: demonstrating value; aligning with local priorities; and collaborating with spoke sites serving rural Veterans. Barriers included: misalignment with local priorities; and hiring delays. COVID was a facilitator, as it stimulated adoption of telehealth practices; and also a barrier, as it consumed attention and resources. Recommendations included: longer startup funding; dedicated funding for human resources to accelerate hiring; funders communicating with local facility leaders regarding how TeleSleep aligns with organizational priorities; hiring into job classifications aligned with market pay; and obtaining, from finance departments, projections and outcomes for the return on investment in TeleSleep.

## 1. Introduction

Sleep apnea, or obstructed breathing during sleep, is a serious public health challenge in the United States, especially among military Veterans (1). In fiscal year 2021, the Veterans Health Administration (VHA) provided care for sleep disorders to 599,966 Veterans, including 189,932 Veterans who accessed this care from their domiciles in rural areas far from major medical centers (2).

To reduce barriers to specialty medical care, VHA developed an Enterprise-Wide Initiative (EWI), referred to as TeleSleep, which uses telemedicine strategies to improve access to sleep care for rural Veterans (3). These strategies include testing Veterans for sleep apnea at their home rather than in a sleep lab; Clinical Video Telehealth applications and other forms of virtual care. The TeleSleep EWI is a hub and spoke model, where larger sites with established Sleep centers provide care for patients referred from smaller, rural sites who do not have sleep medicine programs.

In 2017 and 2020, through its Office of Rural Health (ORH), VHA provided 2 cycles of 3-year start-up funding to launch new TeleSleep programs at rural-serving VA medical facilities, with the desire that participating sites will ultimately sustain their programs with other sources of funding (4).

In late 2021, in order to improve the implementation effort, TeleSleep program leaders [including authors KS, RF, AT, EB, MZ, TR, PC, KW, and MW] decided to explore whether and how funded sites understood and were approaching sustainment. Within the updated Consolidated Framework for Implementation Research (5), funding appears as a construct in both the outer (external to the organization) and inner setting of an intervention. TeleSleep leaders understood ORH start-up funding to be part of the outer setting, with an open question as to which sites would successfully transition to funding from their inner setting.

Researchers have examined sustainment of tele-delivery of innovations ranging from asthma (6) to stroke (7). One study looked at VHA's experiences with sustaining telecare for bipolar disorder (8). While these reports overlap with TeleSleep in their emphasis on innovations, rural health, and telemedicine, including within VHA, we found none that reported on the sustainment of VHA's investments in telehealth delivery of sleep care.

To address this gap in knowledge, in early 2022, an evaluator surveyed the project leader at each of the 24 VA medical facilities that received TeleSleep funding. The survey relied on the Critical Incident Technique as a framework for asking respondents to identify successes, failures, facilitators, and barriers relevant to financially sustaining TeleSleep implementations upon expiration of startup funding (9).

## 2. Methods

### 2.1. Ethics

TeleSleep leaders followed VHA Program Guide 1200.21 and self-certified this inquiry as a non-research, quality improvement activity.

### 2.2. Framework

Between January and September of 2022, TeleSleep leaders met weekly with an evaluator [author JB] who oversaw the evaluation, assisted by author LOD. The evaluation team generally employed informal consensus-seeking techniques to make decisions, continuing discussions until there were no objections to the proposed resolution for the matter at hand.

In its first decision, the evaluation team adopted, as a conceptual framework, the Critical Incident Technique. This technique has evolved over the past 70 years as a method for evaluating purposive (goal-oriented) behavior in individuals and organizations (9, 10). Evaluators using the Critical Incident Technique typically rely on record review, observations, interviews, or surveys to collect data. From such data, evaluators generate insights about five aspects of an individual's or organization's pursuit of a specific purpose: their perception of goals; achievements; failures; success factors; and barriers. Evaluators use this five-point rubric as a deductive framework to structure the overall data collection and analysis, while relying on inductive (grounded) techniques to identify themes within each area. Evaluators can then use the themes to generate requirements and specifications for interventions to improve achievement of the purpose (11, 12).

Through discussion, the TeleSleep evaluation team embraced the Critical Incident Technique as a guiding framework. By late January 2022, the evaluation team agreed on the following evaluation question to serve as the focus of the Critical Incident evaluation: “What are some of the key ingredients for a successful transition from ORH-funded to self-sustaining TeleSleep programs?”

### 2.3. Survey design: domains

In February 2022, the TeleSleep evaluation team discussed data collection strategies. We agreed that a survey would be a productive way to collect data, as we were most interested in the perceptions of key points of contact who represented the sites in the overall TeleSleep program. Sending these key points of contact a survey would allow them to reflect in writing, at their convenience, on our evaluation questions.

On February 18, 2022, the TeleSleep evaluation team agreed on the following survey domains, inspired by our embrace of the Critical Incident Technique framework:

1. How do key stakeholders view the stated goal of sustaining the program beyond ORH funding?
2. Have programs achieved the goal, or feel they are achieving the goal?
3. Have programs failed to achieve the goal, or do they feel they will fall short?
4. What are key success factors that have contributed, or are contributing, to achievement of the goal?
5. What are key barriers that have inhibited, or are inhibiting, achievement of the goal?
6. What other related goals are coming into focus at this stage of the program's development?

### 2.4. Population of ORH-funded TeleSleep sites and administration of survey

In February 2022, TeleSleep leaders provided author JB with a list of 20 sites that received ORH funding to hire personnel as they implemented sleep medicine via telehealth, along with the corresponding key points of contact who would serve as target respondents. In March, author JB emailed a candidate (advanced draft) survey instrument to seven points of contact, for the purpose of obtaining feedback. After minor wording revisions to the survey based on responses from six of the first seven sites surveyed, in April, author JB emailed the revised survey to points of contact for 14 more sites. In early August 2022, TeleSleep leaders realized they had overlooked four sites and JB emailed their points of contact the survey link, and also at this time re-sent the survey to the first-round non-respondent. By August 2022, therefore, author JB had emailed the survey to points of contact qualified to represent the entire survey population of 24 sites. See Supplement for text of survey and summary of revisions. The 24 sites were geographically distributed as follows, using VA's designated catchment areas (13): 5 from the North Atlantic; 2 from the Southeast; 6 from the Midwest; 1 from the Continental; and 10 from the Pacific regions. The 24 sites came from 14 distinct sub-regions known in VA as Veterans Integrated Service Networks.

### 2.5. Interview sample

Following receipt of the surveys, at the direction of the TeleSleep leadership team, author JB also interviewed points of contact at four sites in September 2022. These four sites had responded that they had not achieved sustainment and either were not on track to achieve sustainment or did not answer that question about their trajectory. The interview guide asked them to elaborate on where they were with sustainment. JB recorded the 30 min interviews and took notes and then integrated the notes into the analysis plan as described below.

### 2.6. Analysis plan

Author JB started analyzing survey responses in July 2022 and continued through October 2022, adding interview responses in September. For quantitative analysis, author JB tabulated frequencies of answers on the multiple-choice questions in the survey.

For qualitative analysis, the evaluation team adapted the Critical Incident Technique to our purposes. We had already used the Critical Incident Technique to formulate our survey questions. These questions asked sites to identify goals, achievements, failures, facilitators, and barriers relevant to becoming self-sustaining in TeleSleep. In the analysis phase, we used the Critical Incident Technique as a framework for categorizing responses. While we asked questions in a linear fashion, we knew that respondents might answer laterally, for example citing a barrier when answering a question about facilitators. Therefore, in our analysis, we applied the framework to categorize each response regardless of the question.

To categorize the responses, the evaluation team divided the surveys among four coders (authors JB, AT, LOD, and PC). Each coder analyzed 4–9 surveys, extracting key quotes and coding them as goals, achievements, failures, facilitators, or barriers.

JB's instructions to coders defined the framework categories and provided a worked example for reference. Here are the definitions:

– Goals. Any quote where the respondent points to other goals besides sustainment; can also include any successes, failures, facilitators, and barriers that they reference about these other goals.
– Successes. Any quote where the respondent states they have achieved sustainment or are on track to achieve it.
– Failures/Setbacks. Any quote where the respondent states they have failed to achieve sustainment, or suffered setbacks, or are not on track.
– Facilitators. Any quote where the respondent points to forces in their environment (words, behaviors, events, resources) that propelled the site closer to sustainment.
– Barriers. Any quote where the site respondent points to forces in their environment (words, behaviors, events, resources) that inhibited progress toward sustainment.

JB's instructions also clarified that each coder should expect to categorize excerpts of sentences; that coders could assign the same quote to multiple categories; and that they might assign a quote to a different category than the question category, for example, they might assign the category “barrier” to a quote in a response to a question about facilitators.

When the coders had completed their assignments, author JB clustered the quotes in each category under headings corresponding to themes. During weekly meetings between August and October 2022, the TeleSleep evaluation team discussed and refined the themes. JB then incorporated the interview data and summarized how the themes reflected implicit or explicit recommendations for future improvements in program design. In October, 2022, JB facilitated a discussion of the proposed recommendations at the project's biweekly meeting of TeleSleep site leaders (the survey respondents), revising the recommendations based on input. The site leaders endorsed the recommendations.

## 3. Results

### 3.1. Response rate

All 24 sites surveyed responded between 3/11/22 and 8/17/22. The response rate was 100% and this survey therefore functioned as a census of all ORH-funded TeleSleep sites.

### 3.2. Quantitative or categorical data from surveys and interviews

See Table 1 for a summary of the quantitative and categorical data from our surveys and interviews.

**Table 1:** Summary of quantitative and categorical responses to surveys and interviews.

Location	3-year funding horizon		Year that funding ends					Minimum TeleSleep scope				Sustainment status	
	Aware?	Agree?	20	21	22	23	24	VC	HSAT	Both	Either	Achieved	On track
Midwest 1	0	.		.	.	.	.	1				1	
Southeast 1	1	1			1			1				1	
Midwest 2	1	.				1				1			1
Pacific 1	1	1			1					1		1	
North Atlantic 1	1	1				1			1			1	
Midwest 3	1	0					1			1			1
North Atlantic 2	0	.						.	.	.	.		1
Midwest 4	1	1				1				1			1
Southeast 2	0	0				1				1		1	
Continental 1	0	0		1						1			1
Midwest 5	1	0			1					1		1	
Pacific 2	1	0			1						1	1	
North Atlantic 3	1	1				1					1	1	
Pacific 3	1	1				1				1			1
North Atlantic 4	1	1				1				1		1	
Pacific 4	1	1				1				1		1	
Pacific 5	1	1				1				1		1	
Pacific 6	1	1				1				1		1	
Pacific 7	1	1	.	.	.	.	.			1		1	
Pacific 8	1	1	1							1		1	
North Atlantic 5	1	1				1				1		1	
Pacific 9	1	1					1		1			1	
Pacific 10	1	1				1				1		1	
Midwest 6	1	1				1				1		1	
TOTALS	20	16	1	1	4	13	2	2	2	17	2	18	6

VC, virtual care; HSAT, home sleep apnea testing; “.” denotes no response to the item; 0 denotes “no”; 1 denotes “yes”; blank indicates response not selected.

#### 3.2.1. Achieving sustainment

Eighteen of 24 respondents (75%) said their site had achieved independence from ORH funding, i.e., sustainment. Six more (25%) said they were “on track” to achieve sustainment.

#### 3.2.2. Minimum scope for TeleSleep sustainment

Seventeen of 24 respondents (71%) defined the minimum scope of the TeleSleep program to be sustained as including both Home Sleep Apnea Testing (HSAT) and Virtual Care (e.g., video consultations). Two respondents (8%) defined the minimum scope to be Virtual Care only; two (8%) defined it as HSAT only; and two (8%) defined the minimum scope as either HSAT or Virtual Care. One site (4%) responded that they thought the minimum for sustainment was “virtual BSM [Behavioral Sleep Medicine] services.”

#### 3.2.3. Three year term for ORH funding

Twenty of 24 respondents (83%) indicated their site had entered the project aware of the three-year term of ORH funding and the need to find alternative funding for sustainment. Sixteen respondents (67%) agreed that three years was an appropriate expiration term for the ORH's startup funding; five (21%) disagreed; and three (13%) preferred not to answer this question.

#### 3.2.4. Horizon for ORH funding

One site reported a funding horizon of 2020, and another of 2021. Their funding had expired. Four respondents (17%) represented sites where funding would expire in 2022, thirteen (54%) in 2023, and two (8%) in 2024. Three sites did not respond to this question.

### 3.3. Qualitative data from surveys and interviews

See Table 2 for a summary of qualitative data from our surveys and interviews. Below, we provide a narrative overview featuring examples and quotes.

**Table 2:** Summary of qualitative responses to surveys and interviews.

Achievements	
	Hub-Based Sustainment
	Clinical Resource Hub Sustainment
	Expanded Rural Reach of TeleSleep
	Expanded Non-Rural Reach of TeleSleep
	Innovation
Failures/setbacks	
	Non-Sustainment or Partial Sustainment
	Staff Shortages
Facilitators	
	Supportive Local Administration
	ORH Kickstart and Demonstration of Value
	Hub/Spoke Collaboration
	TeleSleep Procurement of Equipment
	TeleSleep Knowledge Sharing and Moral Support
	COVID Pandemic
Barriers	
	TeleSleep Misalignment with Strategic Goals of Local Leadership
	Friction and Delays in Hiring Process
	Offers Declined Due to Low Pay or Short Employment Horizon
	Complexity of Scheduling for TeleSleep
	COVID Pandemic

#### 3.3.1. Themes related to achieving sustainment

##### 3.3.1.1. Theme: hub-based sustainment

One mechanism for sustainment involved large facilities (or “hub” sites) budgeting for the TeleSleep program as part of their annual operating plans. One respondent described the transition as follows: “At the end of the first 3-year cycle, medical center administration approved the conversion of our temporary TeleSleep positions to permanent positions.”

##### 3.3.1.2. Theme: clinical resource hub sustainment

Some respondents referred to the fact that VA has launched regional Clinical Resource Hubs (CRHs) as a model for telehealth and virtual care. In the VA context, the Sleep CRH model is a regionally-governed program that provides specialty care consultative and chronic disease management services to spoke facilities lacking sufficient resources to meet demand for patient care. A Sleep CRH leverages telehealth, standardized workflows and templates, and sleep middleware to provide consistent high quality and comprehensive sleep care across sites. By engaging with spoke facilities to augment services the CRH creates a sense of community, stabilizing the workforce by reducing burnout and improving retention of spoke practitioners. Transition from a hub-spoke model to a CRH is a natural pathway for hubs located within the same region and represents a larger scale model of shared resources. Several responding sites thought that this model makes sense for sustainment. One respondent stated, “We are now looking at the CRH—or Clinical Resource Hub—to continue our TeleSleep program.”

Another respondent agreed that the CRH could be a viable alternative to local (or hub-based) sustainment and emphasized the need for advance planning either way: “You need to plan at least a year before your term [of ORH funding] ends, whether you're going for CRH or you’re planning for local sustainment.”

In addition, this issue intersected with the concerns respondents had about the slow pace of VA hiring. One respondent, in an interview, said he had the impression that Clinical Resource Hubs were able to hire more quickly and effectively than the ORH project or facilities themselves.

#### 3.3.2. Other achievements

##### 3.3.2.1. Theme: expanded rural reach of TeleSleep

Due to ORH funding of TeleSleep, sites were able to increase their capacity to deliver sleep care remotely and generate more sleep medicine encounters for rural Veterans. One respondent drew attention to the proportion of sleep medicine delivered remotely: “Every sleep clinician now practices TeleSleep at least half of the time. This is a huge change from 2018, partly due to Covid but also partly due to ORH support in staff and equipment (HSAT).” Another reported, “We are about 95% TeleVVC [VA Video Connect, or telehealth to the patient's home] for apnea f/u [follow-up] and have some of the top encounter numbers in VA, for our rural patients.”

Part of VA's logic behind TeleSleep was to reduce referrals to community care, which is a strategic priority for VA in order to reduce costs and increase Veteran satisfaction. One respondent confirmed that this was borne out in practice: “Increase virtual care in order to keep Veterans utilizing VA care.” Another respondent stated, “In [our facility], Sleep has the lowest Care in the Community (CITC) utilization in terms of dollars. Veterans tend to prefer VA care, when available, to community care. Also, Veterans providing positive feedback of having their sleep care managed by VA.”

While telehealth was increasing generally, a respondent indicated that the ORH investment accelerated its uptake: “The ORH grant was instrumental in allowing [our facility] to grow its sleep services, especially to the rural Veterans. This may not have happened without the ORH support.”

VA's logic model for TeleSleep did not anticipate the COVID pandemic. COVID amplified the effects of telehealth for sleep medicine: “When COVID hit it was extremely helpful to have home sleep testing and telehealth already up and going. It was further expanded into telehealth for CPAP set up and follow up.” Sites participating in this network with robust telemedicine services going into the pandemic were able to continue clinical care, particularly in-home video visits, as staffing allowed. Following closure of all in-person sleep testing programs to conserve personal protective equipment, programs with robust HSAT programs were able to leverage these programs to reduce the backlog of patients waiting for sleep testing when services resumed. Projecting into the future, one TeleSleep point of contact also mentioned that this model reduces the potential disruption in healthcare delivery in the event of future catastrophes such as fires, hurricanes, tornados, and other pandemics.

##### 3.3.2.2. Theme: expanded non-rural reach of TeleSleep

Although ORH funded TeleSleep specifically to increase rural Veteran access to sleep medicine, respondents pointed out that many Veterans face distance barriers to accessing care, even in non-rural areas. TeleSleep programs were serving these remote Veterans equally well as rural Veterans. In this context, our evaluation team understood remote to mean facing long distances, while not technically living in an area that was officially classified by the VA as rural. A representative quote on this topic was: “Increasing tele services to Veterans who are remote but not rural.” This demonstrates translation and adaptation of the TeleSleep approach. The TeleSleep initiative indirectly benefits both rural and non-rural Veterans by teaching sleep programs how to implement telemedicine services.

##### 3.3.2.3. Theme: innovation

Respondents defined TeleSleep in terms of core services: Home Sleep Apnea Testing and Virtual Care. They indicated that implementing these services stimulated other innovations. Some of the innovations revolved around delivery of patient services. For example, one respondent wrote about solving the challenge of getting devices returned by mail: “The mailroom told us that they only mail out devices. Mailroom did not provide paid return mailing back to the VA. This is now a daily routine for the mailroom for our HSAT devices.” Another pointed out that the innovations extended beyond pure telehealth services, to include hybrid models of care delivery: “We also started innovative and new programs like Drive Through CPAP pick-ups or Saturday Group CPAP classes.” Other innovations revolved around how to financially account for TeleSleep activities, for example one respondent wrote, “We have also added asynchronous remote PAP monitoring as a capturable workload (prior to COVID, providers would be alerted to downloads as additional signers and would add an addendum, with no workload capture).” Here, capturing workload refers to documenting work in such a way that VHA assigns economic credit to the worker or work unit for the services delivered.

#### 3.3.3. Failure/setback themes

##### 3.3.3.1. Theme: non-sustainment or partial sustainment

Four sites that had initially reported non-sustainment also clarified that sustainment could be partial. Partial sustainment manifested itself in two ways. First, some sites reported only transitioning some of their ORH-funded personnel from term to permanent positions. Second, sites reported that in some cases their ORH-funded personnel, having been hired into permanent positions, were pulled off in other directions unrelated to TeleSleep. One of these sites referred to this as a temporary erosion in capacity for ORH's TeleSleep agenda.

Another respondent indicated that they really had no alternative or backup plan if their hub site did not take over funding of TeleSleep: “I am not certain that our facility leadership will absorb the current staffing that is supporting the TeleSleep Program for both our facility as well our other spoke site. Without the staff whose salaries are being funded by the ORH, the whole Rural TeleSleep program will collapse.”

One respondent pointed to regional variation as to whether hubs or Clinical Resource Hubs might sustain funding for TeleSleep: “It appears each VISN has different needs when it comes to expanding specialty services such as sleep medicine.” Another thought the time horizon was too short: “I think 3 years is too soon to fully build up a program and request institutional funding.” And in the context of so many priorities competing for resources, one respondent concluded: “Sustaining the programs initiated by ORH is a challenge.”

##### 3.3.3.2. Theme: staff shortages

Some sites reported achieving sustainment with the caveat that their TeleSleep programs were understaffed, which they considered falling short of their goals for fully implementing and sustaining the program. Staff were in short supply for all types of employees, ranging from physicians to nurses to sleep technologists. One respondent wrote, “Unable to implement HSAT because of staff shortages.” Another wrote, “We are short staffed on the Sleep Provider side, having only 1 MD [Medical Doctor] who is now part time and retiring in December and 2 Sleep NPs [Nurse Practitioner]. We are also unable to attract and hire the Sleep tech ORH funds were provided for due to the low pay VA pays at GS 8 [8th paygrade in the US government's General Schedule payscale]. I have only 2 Sleep techs and one is now permanently disabled and can only work part time. Our program is badly hobbled by these issues causing us to refer more than half our studies out.” Even upon achieving sustainment, programs that grew from there had to compete like any other program for funds: “Our facility continues to grow and we continue to need more staff. I'm not sure how we'll be able to persuade our Executive Leadership Team to increase our staffing needs.” Our analysis distinguished between programs reporting shortfalls in capacity, which we considered a failure or setback, vs. experiencing friction or delays in hiring, which we discuss in the Barriers section below.

#### 3.3.4. Success factors/facilitators

##### 3.3.4.1. Theme: supportive local administration

Respondents reported that the administrative leadership of the TeleSleep hub sites played a key role in achieving success. Some administrative leaders saw the long-term benefits of hosting and later sustaining a TeleSleep program and were champions in bringing TeleSleep to the hub: “We have a very supportive administration which has been key to success. Many other sites cannot get staff hired or can only hire staff temporarily for the length of the grant. We did not have that trouble and thus were much quicker to get off the ground.” In another case, local administration was supportive but concerned about accepting short-term resources that exceeded what they could sustain in 3 years: “The fact that the funding was only for 3 years was actually cited by our leadership for not accepting all of the FTE [Full Time Equivalent personnel] we were originally awarded.” This site planned for sustainment by committing only in the startup period to what they could sustain longer-term.

##### 3.3.4.2. Theme: ORH kickstart and demonstration of value

In some cases, facility administrative leaders were supportive of sustaining TeleSleep investments from the start. In others, the ORH funding provided time for TeleSleep to demonstrate its value. As one respondent pointed out, “The increased productivity of our sleep program resulting from ORH funding helped gain the support of medical center administration.” Another reported the reaction of local facility leaders: “What are we going to do with all these people that are term [working on short-term contracts]?” And they said, “They’ve all earned their keep. They earned their salary with the workload that they added.”

Another provided more details about the perspective of facility leaders: “Increased interest in TeleSleep by Hub PENTAD [here PENTAD refers to leadership team including: Director of medical center; Associate Director; chief of staff; deputy chief of staff; senior leadership at facility] as it leads to increased unique patients and productivity and availability of funds/devices for home sleep studies.” One respondent emphasized that the demonstration of value required visibility into the data: “Demonstration of adequate encounter volumes and workload at [our facility] to justify conversion of personnel to permanent [made visible through data from QUERI (Quality Enhancement Research Initiative) team].” Another confirmed that TeleSleep was seen as demonstrating value by reducing referrals to community care: “All of the data collected that shows reduction in CITC [Care in the Community] sleep studies directly as a result of VA facilitating home sleep testing has helped us show need for our own sustained sleep program.”

##### 3.3.4.3. Theme: hub/spoke collaboration

VA TeleSleep care is delivered through hubs (larger regional centers) and spoke sites. Spokes include smaller facilities closer to rural Veterans' homes. Respondents indicated that hub/spoke collaboration was a key contributor to TeleSleep success, and ultimately sustainment of this success. On a clinical level, for TeleSleep to succeed, spokes had to accept telehealth practices such as HSAT and Virtual Care [e.g., “Local willingness to use HSAT for diagnosing OSA (Obstructive Sleep Apnea)].”

Then, on an administrative level, hubs and spokes could specify expectations and ultimately count on VA's financial model to provide funds to sites delivering sleep medicine services to Veterans: “We created an MOU [Memorandum of Understanding] with the spoke site outlining a plan for sustainment once ORH funding ended. This included clarification of financial contributions/expectations from each site. We also carefully tracked productivity to show utility of the program.” One interview respondent cited stakeholder engagement in the spokes: “The PENTAD [hub leadership team] does regular town hall meetings with the rural CBOCs [Community Based Outpatient Clinics] because our rural CBOCs are far and they're very vocal, they have a lot of demands.” Stakeholder voices, including those of Veterans accessing TeleSleep, supported sustainment in such settings with local or regional leaders in attendance.

##### 3.3.4.4. Theme: TeleSleep procurement of equipment

Pragmatically speaking, the ORH grant helped programs obtain the equipment and supplies they needed more efficiently than they could have on an individual program basis. ORH was able to organize procurement on a national scale: “National resourcing of testing devices helped with procurement of devices.” Another respondent wrote, “Getting sleep study equipment from ORH grant helped to move the direction of sustainment.”

##### 3.3.4.5. Theme: TeleSleep knowledge sharing and moral support

Respondents reported an intangible but deeply felt success factor: the power of being connected in a network with like-minded peers. This network allowed them to experience the benefits of knowledge sharing (“Leveraging the knowledge and infrastructure to increase teleservices internally”) as well as moral support (“It's been great working with such an awesome support group.”)

##### 3.3.4.6. Theme: COVID pandemic

Respondents cited the pandemic as both a success factor and a barrier (see next section). As a success factor, COVID stimulated more rapid adoption of telehealth (“COVID forced us to implement virtual visits essentially overnight”). The TeleSleep program was well positioned to benefit from a broader organizational commitment to telehealth: “We have been slowly but surely successful in self-sustaining virtual care and portable testing through persistence and necessity (covid)”.

#### 3.3.5. Barriers

##### 3.3.5.1. Theme: TeleSleep misalignment with strategic goals of local facility leadership

Respondents indicated that sometimes they were bringing TeleSleep into an environment where local leaders did not see the relevance of the program to their organization's strategic goals. One respondent characterized the dynamic as follows: “Basically there was no overt support from the local facility leadership with re: ORH [TeleSleep] program—if it was not contributing to the local leadership metrics.” One respondent pointed to local leaders pulling TeleSleep personnel into other initiatives: “My MSA [Medical Support Assistant] is now pulled because we no longer have her dedicated to us. And so now she's pulled to other areas too and trying to still cover for us…. So my HSAT person, she may be called to do an emergent EKG [electrocardiogram] just because she has experience doing EKGs and she's the only person here.” Others pointed to conflicting goals, such as capping hires vs. recapturing VA care being referred to community providers: “In many other facilities there is a disconnect between not hiring above an FTE cap and bringing back Community Care dollars into the VA.”

##### 3.3.5.2. Theme: friction and delays in hiring process

Hiring, a centralized function in VA, was seen as slow: “But one of the main limitations for us is the lack of HR [Human Resources department] assistance/support during hiring. I actually had to find my own staff and ended up doing direct hiring. Even this took about 1 year to actually hire the staff from outside the VA, and over 6 months for a transfer of a VA employee from one facility to another. This was very frustrating. It was very easy to lose potential staff due to the long interval for HR to actually hire a new employee.”

Some of the delays were due to long request and approval processes: “But what I normally have to do is go through my medical service chief, who has to do an RMC [Resource Management Committee] request that goes to a board, who then has to after that's approved, it goes to the resource management board and then it goes on for to be certified and signed off by the Center Director and the PENTAD. But you know, there's no transparency, you never know where anything is in the process.”

Hiring delays were especially painful in the context of a short ORH funding horizon of three years. In addition, there was only a short lead time between approval of funding and the start of the project. This meant that sites did not have lead time in which to be posting jobs and working with Human Resources. As one respondent wrote, “In the research world, what happens is you know you're going to get funding well in advance of receiving the funds. But in this case, you really don't have much lead time.”

Furthermore, sites could not carry over unexpended funds, so the lead time required for hiring ultimately led to money being returned unspent. One interview respondent indicated that she didn't know the extent of support that could be requested until the second year and so missed out on a year of scheduling support: “I had no idea that I could ask for an administrative person, which we didn't have. And so then I learned that when I went to the Office of Rural Health kickoff, and so the next year I asked for quite a bit more. And we were able to get that MSA [Medical Support Assistant]. That was hugely helpful.”

##### 3.3.5.3. Theme: offers declined due to pay or short employment horizon

Problems with hiring were often due to the complex and lengthy hiring process at VA, but sometimes were due to other structural issues. Specifically, some of the jobs posted could be unappealing in terms of having short horizons: “It is so hard to get approval to hire individuals with a guarantee from the service line that funds will be sustained by the local facility after ORH funds. In other words, it is tough to tell a person interviewing for a job that it may not be there 6 or 8 months after they start if the service line cannot sustain the position.” Others had lower-than-market pay rates: “The last guy that expressed some interest lost interest [in a sleep tech position] when I told him what the pay was [GS-8] [General Schedule or government paygrade 8] in the 20s [20 dollars per hour wage rate]. He had another job offer making 40 an hour, which is equal to GS 12 [General Schedule or government paygrade 12] step one.”

##### 3.3.5.4. Theme: complexity of scheduling for TeleSleep

Respondents indicated that scheduling for TeleSleep is complex and requires 80 h of training in VA and an understanding of complex sleep testing and clinical workflows. Experienced schedulers are hard to find: “Difficulties with staffing of MSAs [Medical Support Assistants] to schedule VVC [VA VideoConnect, or telehealth to patient home] patients. Even with experienced MSAs, scheduling for telehealth sleep care is hard. The scheduling program was designed 30 years ago for face-to-face appointments. In addition, there are different appointments that fall in the category of telehealth. One respondent said this created confusion: “Poor understanding at both hub and spoke of differences in scheduling VVC [VA VideoConnect, or telehealth to patient home telehealth to patient home ] vs. CVT [Clinical Video TeleHealth or telehealth between VA facilities] appointments.”

##### 3.3.5.5. Theme: COVID pandemic

As noted earlier, respondents cited the pandemic as both a success factor and a barrier. While COVID accelerated the adoption of telehealth, the crisis consumed VA resources and impeded progress for TeleSleep as well as many other initiatives. One respondent wrote, “My experience with ORH ran through the pandemic. I think 3 years may well be adequate at another time. However, dealing with the pandemic was such a daily challenge and focus at our facility, that 3 years was not near enough time for our program to develop the traction needed for sustainment.”

## 4. Discussion

The point of a Critical Incident Technique survey is to generate insights that lead to improvements. Responses to our survey did specify, explicitly or sometimes implicitly, conditions for improvement. Through discussion among TeleSleep leaders, we synthesized the following recommendations for each major audience, based on the survey findings.

### 4.1. Recommendations

#### 4.1.1. Recommendations for leaders of the TeleSleep enterprise-wide initiative (provider of startup funding for TeleSleep)

TeleSleep local site leaders made apparent the need for greater clarity around expectations of timelines with respect to hiring, initiating services, and sustainment planning. Additionally, creation of other toolkits such as one that supports hiring would be helpful (i.e., a toolkit with a broad range of functional statements, position descriptions, and proposals for special salary rates). In addition, providing sample business cases to support individual site leaders' attempts to garner support for conversion of temporary staff to permanent positions would have been helpful.

#### 4.1.2. Recommendations for the office of rural health—funder and instigator of the TeleSleep enterprise-wide initiative

The first recommendation was for the ORH to consider longer project horizons. VA sites face friction and delays in hiring, so three years represents only 1–2 years of actual implementation after accounting for hiring. A related recommendation was to provide more lead time between approving a project and its start date. This would allow project leaders time to get themselves organized for implementation of their proposed project plan.

Respondents were sympathetic with the many demands on centralized Human Resources functions but were concerned about how long hiring took. TeleSleep leaders recommended that funders consider providing extra funding or capacity for Human Resources to have capacity to recruit and hire personnel for funded projects.

TeleSleep also recommended that funders require a proposed sustainment plan, perhaps with letters of support, prior to approving expenditures. One respondent felt the funder could also do more to facilitate coordination with the VA national program office for sleep medicine; the TeleSleep programs around the country; and the administrative leadership at each facility hosting a TeleSleep program. For example, one idea is for the funder to facilitate better recognition of the alignment between national and local priorities.

While survey respondents applauded the helpfulness of the TeleSleep leadership team, they also expressed a request for a playbook or reference manual from the funder.

#### 4.1.3. Recommendations for sleep medicine leaders at each funded site—implementers of the TeleSleep program

TeleSleep leaders felt that one lesson learned was for sleep medicine leaders to more mindfully align their innovation agenda with the strategic goals of facility leaders. This could include writing a business case anticipating increases in clinical volumes or recapture of community care. Implementers could do more to communicate the business case to regional, hub, and spoke stakeholders. The business case would need to emphasize not just financial benefits but also improvement in Veteran-centered care.

Based on the experience of survey respondents, TeleSleep leaders recommended that TeleSleep implementers measure and report the early demonstrations of value from their project and begin planning for sustainment immediately through alliances with hub sites, spoke sites, or Clinical Resource Hubs.

Among the lessons learned from the survey respondents was for implementers to begin hiring as soon as possible and expect friction and delays. Also, to work around hiring barriers with a strategic eye to what a local site needs; who is available locally; and what the market is. Specifically, TeleSleep implementers should consider hiring respiratory therapists who can perform functions of sleep technicians such as conducting sleep studies because the gap in market pay may be less for respiratory therapists than it is for sleep technicians. Implementers should also consider using hiring and retention bonuses when available to overcome salary gaps compared to private-sector market conditions; and communicate the full value of VA employment, including the financial value of the benefits.

Implementers should consider stakeholder engagement, such as asking Veterans to speak at town hall meetings if they feel their access has improved due to program innovations. This can attract the support of local or regional leaders.

#### 4.1.4. Administrative leaders at VA facilities with funded TeleSleep programs

TeleSleep leaders felt that an innovation like their program was clearly in line with VA national priorities (e.g., improving access to care) and senior executive service metrics (e.g., utilizing specialty care telemedicine at various thresholds). Therefore, their request for administrative leaders at local VA facilities was to consider aligning their local agenda with national initiatives and embrace innovations that advance Veteran care. Their advice was to pay attention to efforts by programs like TeleSleep to improve performance on metrics, such as care in the community, before those metrics reflect a crisis. Their feeling was that too often, facility leaders wait until a problem reaches crisis conditions to invest.

One counter-intuitive recommendation for sustainment of innovations was for early implementers to not accept amounts of funding that could not be sustained in the longer term. One site in our survey took less funding, and hired fewer people, because they knew in advance what they would be able to sustain.

#### 4.1.5. VA Human Resources

In line with the earlier recommendation for funders to provide extra capacity to human resources, TeleSleep leaders suggested that this would allow human resources to expedite hiring for innovative programs with short-term funding so they can get started promptly. TeleSleep leaders also suggested that VA's centralized human resources departments consider revising position descriptions and job classifications so that pay and benefits are better aligned to match market conditions.

#### 4.1.6. VA finance

TeleSleep leaders had two requests or recommendations for VA's finance functions. First, these functions ideally would provide projections of future impact on VA funds allocation for new programs such as TeleSleep. Second, the finance department should ideally report on how the new programs perform against the projections. This would allow program stakeholders to see the return on their investments.

#### 4.1.7. Regional leadership

TeleSleep leaders felt that the Veteran Integrated Service Networks (VISNs), or VA regions, should increase consideration of approaches for regional pooling of resources and shared services, whether through a Clinical Resource Hub or other mechanisms.

### 4.2. Connections to the literature

VHA scientists recently surveyed representatives of 49 sites six months after expiration of their organizational support to implement evidence-informed practices through a program called Diffusion of Excellence (14). Their survey, with a response rate of 64%, found that 71% had sustained the innovations. Three of the 45 innovations related to telemedicine: TeleWound care; Video Blood Pressure Visits; and Advanced Comprehensive Diabetes Care. None of the innovations related to tele-delivery of sleep medicine.

Our census of TeleSleep sites found a higher forecasted rate of sustainment but was more exploratory than summative: we asked our questions of 22 sites whose funding had not yet expired, so we cannot be sure if these sites will in fact achieve sustainment upon expiration of their funding. Further evaluation could retrospectively compare the rate of TeleSleep sustainment to the rate found for Diffusion of Excellence innovations at VHA.

Similar to our survey, the Diffusion of Excellence study found that COVID was both a facilitator, in that it accelerated adoption of virtual care, and a barrier, in that some COVID guidance interrupted implementation of some programs. Like us, the Diffusion of Excellence team identified the need to report on partial sustainment, rather than strictly dichotomizing sustainment into a yes/no outcome. Their survey also found that staffing shortfalls interfered with sustainment; and delays in hiring or reassigned staff were barriers to sustainment. The response they cite on this topic precisely mirrors several that we received: “One of our positions has been vacant since January and the other position was realigned under a specific specialty care service.”

While the Reardon study broadly examined 45 disparate innovations at VHA, Bauer and colleagues reported in depth on one VHA telemedicine initiative that has some similarities with TeleSleep: the Bipolar Disorders Telehealth Program (8). In this telehealth program, VHA provides funding for the implementation of Clinical Video Teleconferencing (CVT). This is the same telehealth intervention that TeleSleep uses for virtual delivery of sleep medicine to patients in VA clinics. The Bipolar Telehealth study examined quantitative measures relating to Reach, Efficacy, Adoption, Implementation, and Maintenance; and qualitative measures relating to the innovation, recipients, inner and outer context, and facilitators of implementation.

A key feature of the outer context was the “provision of centralized VA funding and resources to make Bipolar Telehealth available to facilities without the expectation that individual facilities provide financial support.” In contrast, our evaluation asked sites about if and how they were able to sustain TeleSleep after the expiration of a defined three-year period of startup funding from the Office of Rural Health.

Bauer and colleagues found steady growth over time in consults among 35 participating facilities as evidence of sustainment. Many facilities did make in-kind investments of telehealth space, equipment, and support staff. In common with our findings, the Bipolar Telehealth program also reported complexity of scheduling and availability of staff as barriers, while common facilitators included delivery of care valued by Veterans and smooth collaboration between their central hub and the participating sites. Further research is needed to determine whether sites would financially sustain the telemental health program in the absence of centralized VA funding.

### 4.3. Limitations

The main limitation associated with this report is that it was a Quality Improvement project conducted within the Veterans Health Administration. Other systems implementing telehealth for sleep medicine may face different dynamics related to sustainment. Therefore our findings may not generalize to other settings.

In addition, while we surveyed the implementing sites about their views of sustainment, we did not query other stakeholders. The implementing sites generated recommendations for a specific set of stakeholders: the funder; clinical leaders; administrative leaders; the Human Resources department; the Finance department; and regional leaders. Future studies should follow up with these stakeholders to obtain their views. Among the stakeholders we did query, we relied entirely on self-report. This may have introduced biases, such as social desirability, to our findings.

## 5. Conclusions

Our evaluation employed a qualitative framework, the Critical Incident Technique, to generate field-based recommendations for improvement in how stakeholders, including funders, champions of the innovation, and local administrative leadership, can facilitate the transition from external to internal financing of an evidence-based innovation. The lessons learned from this sustainment survey exercise extend beyond TeleSleep and may be applicable to similar enterprise-wide initiatives within VA and other health delivery systems.

## Data Availability

The raw data supporting the conclusions of this article will be made available by the authors, without undue reservation.
