# Supplementary material for: Sustainment of the TeleSleep program for rural veterans
Source: Front Health Serv. 2023 Nov 10;3:1214071. doi: 10.3389/frhs.2023.1214071 (PMC10668014; doi:10.3389/frhs.2023.1214071)
Supplement: Supplementary file 1 [file Datasheet1.docx]

## Supplement: Survey Design and Content

Author JB drafted survey items corresponding to the survey domains and sent version 1 of the survey to seven site points of contact who were also on the TeleSleep leadership team. Six of the seven responded promptly and provided feedback on the survey items along with their responses. Author JB made minor revisions, creating the second and final version of the survey, shown below, with notes indicating revised items.

**TeleSleep Survey on Sustainment – Second and final version**

*“This survey is for key points of contact at sites that have ORH TeleSleep program funding for full-time equivalent personnel. The survey explores your views regarding the ORH's stated goal of transitioning from ORH-funded to self-sustaining TeleSleep programs after a maximum of 3 years of ORH support. There are 10 required questions, 7 of which are multiple choice, and respondents have been taking 5-10 minutes to complete.”*

**1/2. Please respond to this survey on behalf of a site that receives (or received) ORH TeleSleep program funding for full-time equivalent personnel.**

The survey will refer to that site as your designated site. Which site would you like to designate for this survey submission?

[List of 24 sites in a drop-down.]

Please use the space below to elaborate on your response to the question above.

**3/4. When your designated site joined the TeleSleep program, were you aware of the ORH's goal of transitioning from ORH-funded to self-sustaining TeleSleep programs after a maximum of 3 years of ORH support?**

Please answer yes, no, or prefer not to answer, and then use the space below to elaborate/clarify your response.

**5/6. Do you agree with the ORH's goal of transitioning sites to self-sustaining status after a maximum of three years of ORH funding?***

Please answer yes, no, or prefer not to answer, and then use the space below to elaborate/clarify your response.

**7. In what year will your site's ORH funding expire?****

[Dropdown featuring list of fiscal years from 2020 to 2025, along with “I don’t know” and “prefer not to answer”]

**8. Please use the space below to elaborate on your response to the question above.**

**9. In your view, what is the minimum scope of TeleSleep services that a site would have to sustain in order to show that it had successfully transitioned to self-sustaining?*****

Virtual Care only is the minimum

Home Sleep Apnea Testing only is the minimum

Virtual Care and Home Sleep Apnea Testing together are the minimum

Prefer not to answer

**10. Please use the space below to elaborate on your response to the question above.**

**11/12. By your definition of success above, has your designated site already achieved the goal of transitioning to self-sustaining status of TeleSleep?**

Please answer yes, no, or prefer not to answer, and then use the space below to elaborate/clarify your response.

**13/14. By your definition of success above, is your designated site on track to achieve the goal of transitioning to self-sustaining status of TeleSleep before the expiration of your three-year maximum time horizon for ORH funding?**

Please answer yes, no, or prefer not to answer, and then use the space below to elaborate/clarify your response.

**15. What have been some of the key facilitators contributing to any progress in your designated site's quest for self-sustaining TeleSleep?**

Please list any broad factors or forces or events in the environment, as well as specific actions or work products undertaken by individuals or organizations, which have moved you in the direction of self-sustaining TeleSleep.

**16. What have been some of the key barriers hindering progress in your designated site's quest for self-sustaining TeleSleep?**

Please list any broad factors or forces or events in the environment, as well as specific actions or work products undertaken by individuals or organizations, which have moved you away from self-sustaining TeleTSleep.

**17. What are new goals or achievements that have come into focus for your designated site, in relation to delivering sleep care to Veterans, as a result of participating in the TeleSleep program?**

**18. Please provide any other comments or feedback regarding the contents of this survey**

* The first version of this item asked, “Do you feel the ORH’s stated goal of transitioning sites to self-sustaining status is appropriate?”

** The first version of this item asked, “When did or when will your site's three-year horizon for ORH funding end?”

*** The first version of this item stated, “Sites may differ in their definition of ‘successful transition to self-sustaining TeleSleep programs.’ In your view, what scope of TeleSleep does your site have to sustain in order to judge that it had successfully transitioned to self-sustaining?
